# Low Amount of Salinomycin Greatly Increases Akt Activation, but Reduces Activated p70S6K Levels

**DOI:** 10.3390/ijms140917304

**Published:** 2013-08-22

**Authors:** Ju-Hwa Kim, Ae-Ran Choi, Yong Kee Kim, Hyung Sik Kim, Sungpil Yoon

**Affiliations:** 1 Research Institute, National Cancer Center, Ilsan-gu, Goyang-si, Gyeonggi-do 410-769, Korea; E-Mails: jua@ncc.re.kr (J.-H.K.); 73373@ncc.re.kr (A.-R.C.); 2 College of Pharmacy, Sookmyung Women’s University, Seoul 140-133, Korea; E-Mail: yksnbk@sookmyung.ac.kr; 3 School of Pharmacy, Sungkyunkwan University, Suwon 440-746, Korea; E-Mail: hkims@skku.edu

**Keywords:** Salinomycin, Akt, p70S6K, mTOR, LY294002, wortmannin, cancer

## Abstract

The present study identified a novel salinomycin (Sal)-sensitization mechanism in cancer cells. We analyzed the signal proteins Akt, Jnk, p38, Jak, and Erk1/2 in cancer cell lines that had arrested growth following low amounts of Sal treatment. We also tested the signal molecules PI3K, PDK1, GSK3β, p70S6K, mTOR, and PTEN to analyze the PI3K/Akt/mTOR pathway. The results showed that Sal sensitization positively correlates with large reductions in p70S6K activation. Interestingly, Akt was the only signal protein to be significantly activated by Sal treatment. The Akt activation appeared to require the PI3K pathway as its activation was abolished by the PI3K inhibitors LY294002 and wortmannin. The Akt activation by Sal was conserved in the other cell lines analyzed, which originated from other organs. Both Akt activation and C-PARP production were proportionally increased with increased doses of Sal. In addition, the increased levels of pAkt were not reduced over the time course of the experiment. Co-treatment with Akt inhibitors sensitized the Sal-treated cancer cells. The results thereby suggest that Akt activation is increased in cells that survive Sal treatment and resist the cytotoxic effect of Sal. Taken together; these results indicate that Akt activation may promote the resistance of cancer cells to Sal.

## 1. Introduction

Salinomycin (Sal) was originally used to eliminate bacteria, fungi, and parasites [1–3]. More recently, this drug has been exploited to inhibit the growth of tumor stem cells and chemoresistant cancer cells [4–12]. Sal also functions as an efflux pump p-glycoprotein (P-gp) inhibitor [13–15] and is considered to be a potential anti-cancer drug for cancer chemoprevention. Additionally, Sal sensitizes cancer cells to the effects of doxorubicin, etoposide, radiation, and anti-mitotic drugs, thereby inducing apoptosis as a result of DNA damage and reduced p21 protein levels due to increased proteasomal activity [14,16,17]. A more complete understanding of the mechanisms governing Sal sensitization is required to facilitate the therapeutic use of Sal in patients with cancer.

Identification of the proteins that have altered expression and activity following Sal treatment will yield valuable clues concerning the mechanisms involved in Sal-induced apoptosis and resistance. As the activation and inactivation of signaling kinases contributes to drug-induced apoptosis and chemo-resistance associated with cancer [18–22], it is important to determine which signaling kinases are altered when cells are exposed to Sal. Signaling kinases relevant to drug-induced apoptosis or resistance include the critical proteins Akt, Jnk, p38, Jak, Erk1/2, and c-Src [18–25].

Here, we investigated changes in the expression and activation of growth signaling kinases and principal signaling proteins in Sal-treated cancer cells. We report that Sal sensitizes cancer cells by reducing the p70S6K activation, but activates Akt. The Akt activation contributes to the reduction of Sal-induced apoptosis. This novel finding regarding Sal-sensitization mechanisms could facilitate the therapeutic use of Sal in patients with cancer.

## 2. Results and Discussion

### 2.1. Low Concentration of Sal Highly Activates Akt

We investigated the proteins altered by Sal in the Hs578T breast cancer cell line, which has been well-studied in previous studies [14,16,17,26]. In particular, we sought to identify important proteins that were altered by relatively low Sal concentrations. This was important as clinical applications will require relatively low concentrations of Sal to avoid toxicity and harm to normal cells. The low Sal concentrations 0.5 μM was selected based on the results from our previous study [26]. We assessed the influence of Sal on the activation status and levels of Akt, Jnk, p38, Jak, Erk1/2, Jak1, Jak2, c-Src, PI3K, and IKKα/β, which are pivotal factors in the major signaling pathways regulating cell growth [18–25]. We also tested their activation status and protein levels at both early (12 h) and late (24 h) time points after Sal treatment, to observe the changes in a time-dependent manner.

**Figure 1:**
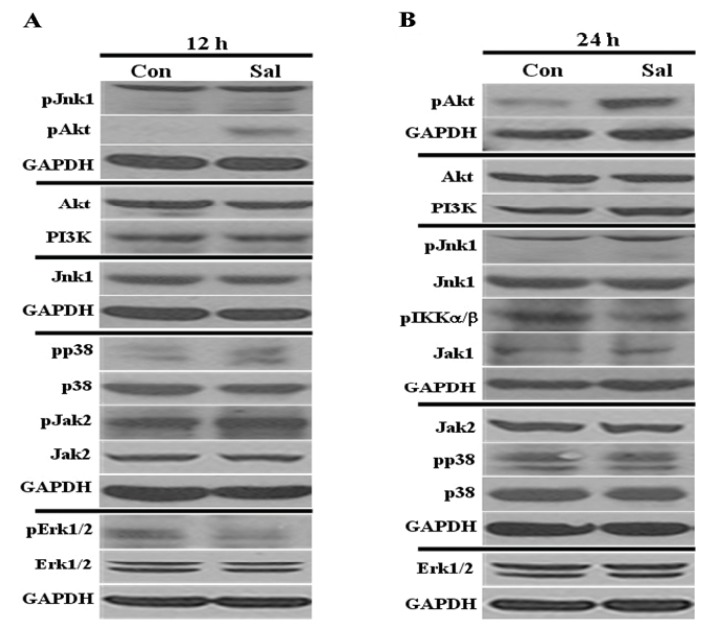
Low concentration of Sal highly activates Akt. Hs578T cell extracts were collected at (**A**) 12 h and (**B**) 24 h after treatment with 0.5 μM Sal or from Dimethylsulfoxide (DMSO)-treated samples (Con). Western blot analyses were performed using antibodies against pJnk1, pAkt, Akt, PI3K, Jnk1, pp38, p38, pJak2, Jak2, pErk1/2, Erk1/2, pIKKα/β, Jak1, and glyceraldehyde 3-phosphate dehydrogenase (GAPDH).

As shown in Figure 1, Sal treatment did not alter the total levels of these proteins at 12 h and 24 h. We then observed and analyzed active forms of these signal proteins. We found that Akt activation occurred at the earlier time point (12 h) and was increased at the later time point (24 h). Phosphorylated Jnk1, p38, Erk1/2, IKKα/β, and Jak2 were detected in the control cells at 12 h or 24 h, but these levels showed negligible changes and were unaffected by Sal treatment (Figure 1). Phosphorylated forms of PI3K, c-Src, and Jak1 were not detected in the control or Sal-treated cells (data not shown). Taken together, we found that only Akt was significantly activated at both 12 h and 24 h, thereby indicating that the inhibition of cellular proliferation by Sal positively correlates with increased Akt activation.

### 2.2. Sal Reduces Levels of Phosphorylated P70S6K, Survivin, and FOXO1

**Figure 2:**
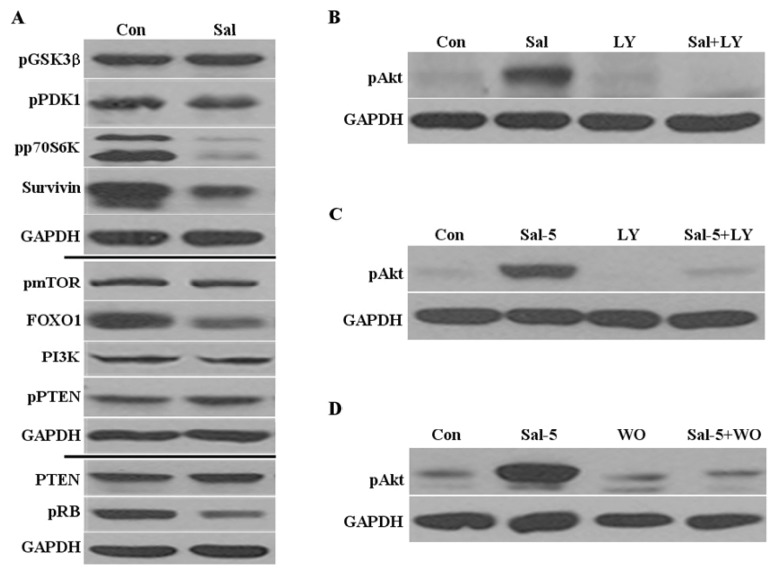
Akt activation by Sal is dependent on the PI3K pathway. (**A**) Hs578T cell extracts were collected 24 h after treatment with 0.5 μM Sal or from DMSO-treated samples (Con). Western blot analyses were performed using antibodies against PI3K, pPDK1, pGSK3β, pp70S6K, pmTOR, pPTEN, PTEN, survivin, FOXO1, pRb, and GAPDH; (**B**–**D**) Hs578T cells were plated on 60 mm-diameter dishes and grown to 30%–40% confluence. The cells were then stimulated for 24 h with 0.5 μM Sal (Sal), 5 μM Sal (Sal-5), 20 μM LY294002 (LY), 1 μM wortmannin (WO), 0.5 μM Sal with 20 μM LY294002 (Sal + LY), 5 μM Sal with 20 μM LY294002 (Sal-5 + LY), 5 μM Sal with 1 μM wortmannin (Sal-5 + WO), or DMSO (Con). The cells were used for western blot analysis using antibodies against pAkt and GAPDH.

Since Akt activation is involved in proliferation and survival signals [24], we further analyzed whether Sal influences the activation status or levels of the signal proteins that function upstream and downstream of Akt activation. In this study, we tested the major proteins PI3K, PDK1, GSK3β, p70S6K, mTOR, and PTEN, which are involved in the PI3K/Akt/mTOR pathway. We did not detect any significant alterations to the activation statuses or protein levels of the analyzed proteins, with the exception of phosphorylated p70S6K, which was significantly reduced (Figure 2A). We assumed that Sal-sensitization involves the effective reduction of p70S6K activity, which is a major target for inhibiting the mTOR pathway [27]. We also tested the downstream targets survivin, which is a tumor suppressor protein, and its transcriptional regulator, FOXO1 [27]. We found that Sal reduced the levels of both survivin and FOXO1 (Figure 2A), thereby suggesting that Akt activation does not influence the downstream targets. As previously demonstrated [16], we observed that the pRb levels were reduced by Sal (Figure 2A). The results suggest that Akt activation by Sal does not influence the activation of other tested proteins in PI3K/Akt/mTOR pathway. Although Sal-sensitization increases Akt activation, we assumed that Sal reduces the proliferation signals of the mTOR pathway by reducing the levels of phosphorylated p70S6K, survivin, and FOXO1.

### 2.3. Akt Activation by Sal Requires the PI3K Pathway

Although we did not detect any changed upstream and downstream targets of Akt activation, we further tested how Akt activation by Sal occurs. To determine whether the Akt activation by Sal is required by the PI3K pathway, cells were co-treated with Sal and LY294002, which is a specific PI3K pathway inhibitor [27,28]. Activation of Akt was abolished by this co-treatment (Figure 2B). The effect of LY294002 treatment on Akt activation was strong and even the high levels of activated Akt induced by 5 μM Sal were abolished by the LY294002 co-treatment (Figure 2C). We used another specific PI3K pathway inhibitor, wortmannin [27], to confirm that Akt activation by Sal depends on the PI3K pathway (Figure 2D). These observations are consistent with the suggestion that the PI3K pathway is required for Sal-mediated Akt activation.

### 2.4. Akt Activation by Sal Correlates with Increased Cellular Apoptosis

**Figure 3:**
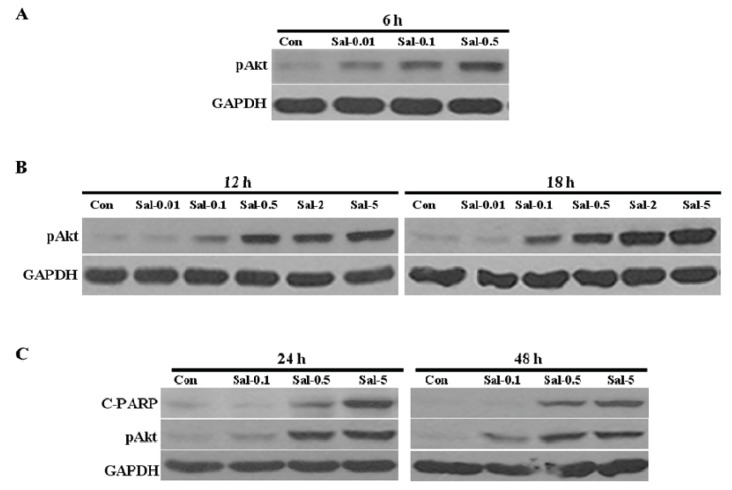
Akt activation by Sal correlates with increased cellular apoptosis. (**A**–**C**) Hs578T cells were plated on 60 mm-diameter dishes and grown to 30%–40% confluence. The cells were then stimulated for 6, 12, 18, 24, or 48 h with 0.01 μM Sal (Sal-0.01), 0.1 μM Sal (Sal-0.1), 0.5 μM Sal (Sal-0.5), 2 μM Sal (Sal-2), 5 μM Sal (Sal-5), or DMSO (Con). The cells were used for Western blot analysis using antibodies against pAkt, C-PARP, and GAPDH.

To measure the relationship between the level of Akt activation and Sal toxicity, we analyzed the time-dependent and dose-dependent effects of this drug on Akt activation. The increased Akt activation was detected at six hours after Sal treatment (Figure 3A), thereby suggesting that the Akt pathway is involved very early. The Akt activation positively correlated with Sal concentration (Figure 3A–B), thereby suggesting that Akt activation by Sal positively correlates with increased cellular apoptosis. When we directly compared Akt activation and C-PARP production, we found similar increased patterns with dependence on dose of Sal treatment (Figure 3C). In addition, the increased levels of pAkt were maintained over the time course of the experiment. The observations in this study are consistent with the suggestion that increased apoptosis following Sal exposure positively correlates with increased Akt activation.

### 2.5. Sal-Mediated Akt Activation Is Conserved in Other Cell Lines

**Figure A1:**
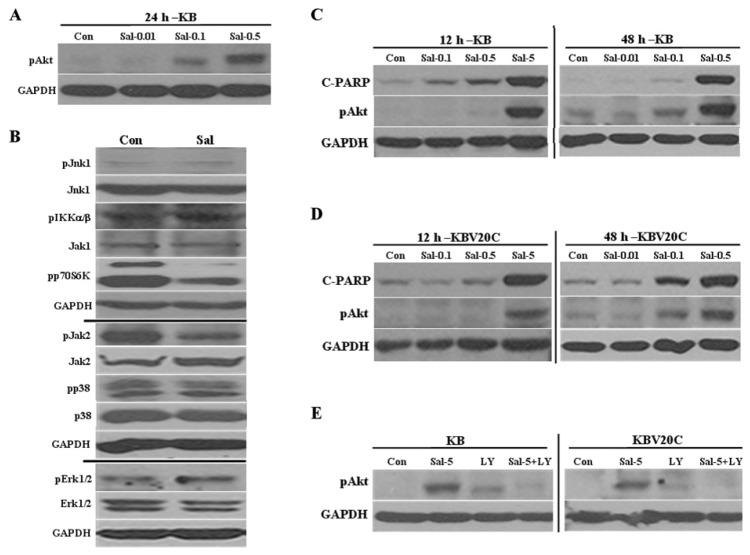
The Sal-mediated Akt activation is conserved in other cell lines. (**A**) KB cell extracts were collected 24 h after treatment with 0.01 μM Sal (Sal-0.01), 0.1 μM Sal (Sal-0.1), 0.5 μM Sal (Sal-0.5), or DMSO-treated (Con). Western blot analyses were performed using antibodies against pAkt and GAPDH. (**B**) KB cell extracts were collected 24 h after treatment with 0.5 μM Sal (Sal) or from untreated samples (Con). Western blot analyses were performed using antibodies against pJnk1, Jnk1, pErk1/2, Erk1/2, p38, pp38, pJak2, Jak1, Jak2, pIKKα/β, pp70S6K, and GAPDH. (**C**,**D**) KB and KBV20C cells were plated on 6-well plates and grown to 30%–40% confluence. The cells were then stimulated for 12 or 48 h with 0.01 μM Sal (Sal-0.01), 0.1 μM Sal (Sal-0.1), 0.5 μM Sal (Sal-0.5), 5 μM Sal (Sal-5), or DMSO (Con). The cells were used for western blot analysis using antibodies against C-PARP, pAkt, and GAPDH. (**E**) KB and KBV20C cells were plated on 6-well plates and grown to 30%–40% confluence. The cells were then stimulated for 24 h with 5 μM Sal (Sal-5), 20 μM LY294002 (LY), 5 μM Sal with 20 μM LY294002 Sal-5 + LY), or DMSO (Con). The cells were used for western blot analysis using antibodies against pAkt and GAPDH.

We further tested whether Sal treatment increases Akt activation in other cancer cell lines originating from a different organ. The oral squamous cancer cell line, KB was tested for Akt activation by Sal. As seen in Figure A1A,B, Sal increased the Akt activation at 24 h, whereas the phosphorylated forms of the other signaling molecules were not detected. As seen in the Hs578T cells, we also detected large reductions in p70S6K activation (Figure A1B). These results indicate that the roles of Akt and p70S6K in the PI3K/Akt/mTOR pathway are conserved in cancer cells that originate from different organs.

As seen in the Hs578T cells, both the Akt activation and C-PARP levels were dependent on concentration of Sal (Figure A1C), thereby suggesting that Akt activation by Sal positively correlates with increased cellular apoptosis. We also tested KB’s multidrug-resistant subline, KBV20C [29], to observe whether Akt activation by Sal depends on multi drug-resistance (MDR). As seen in Figure A1D, the KBV20C cells responded similarly to the sensitive cell line KB at both 12 h and 48 h. The above results indicate that the Akt activation is independent of the MDR phenotype. The Akt activation was also abolished by LY294002 (Figure A1E), thereby suggesting that the PI3K pathway is also required for Sal-mediated Akt activation in these cell lines. Together, the results suggest that the Sal-mediated increased Akt activation is conserved in cancer cells that originate from different organs.

### 2.6. Co-Treatment with an Akt Inhibitor Increases Apoptosis of Sal-Treated Cells

**Figure 4:**
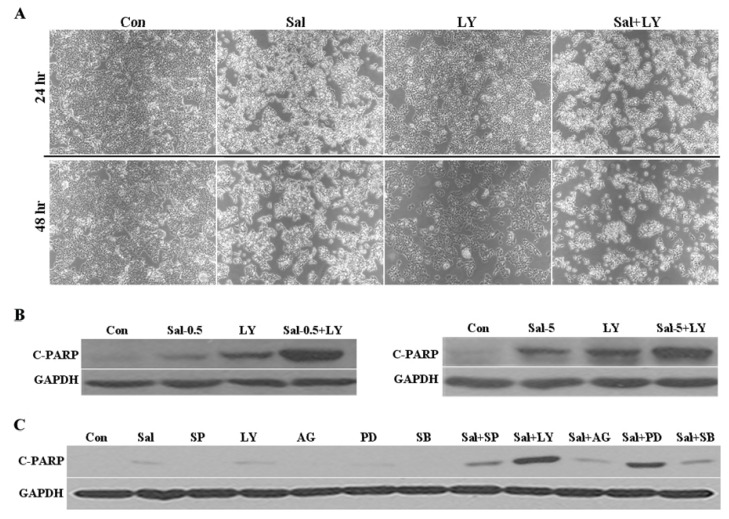
Co-treatment with Akt inhibitor increases apoptosis of Sal-treated cells. (**A**) Hs578T cells were grown on 6-well plates and treated for 24 h with 0.5 μM Sal (Sal), 20 μM LY294002 (LY), 0.5 μM Sal with 20 μM LY294002 (Sal + LY), or DMSO (Con). After 24 h and 48 h (B), the cells were observed using an inverted microscope with a 5× objective lens; (**B**) Hs578T cells were plated on 60 mm-diameter dishes and grown to 30%–40% confluence. The cells were then stimulated for 24 h with 0.5 μM Sal (Sal-0.5), 5 μM Sal (Sal-5), 20 μM LY294002 (LY), 0.5 μM Sal with 20 μM LY294002 (Sal-0.5 + LY), 5 μM Sal with 20 μM LY294002 (Sal-5 + LY), or DMSO (Con). The cells were used for Western blot analysis using antibodies against C-PARP and GAPDH; (**C**) Hs578T cells were plated on 60 mm-diameter dishes and grown to 30%–40% confluence. The cells were then stimulated for 24 h with 0.5 μM Sal (Sal), 20 μM SP600125 (SP), 20 μM LY294002 (LY), 20 μM AG490 (AG), 50 μM PD98059 (PD), 10 μM SB203580 (SB), 0.5 μM Sal with 20 μM SP600125 (Sal + SP), 0.5 μM Sal with 20 μM LY294002 (Sal + LY), 0.5 μM Sal with 20 μM AG490 (Sal + AG), 0.5 μM Sal with 50 μM PD98059 (Sal + PD), 0.5 μM Sal with 10 μM SB203580 (Sal + SB), or DMSO (Con).

**Figure A2:**
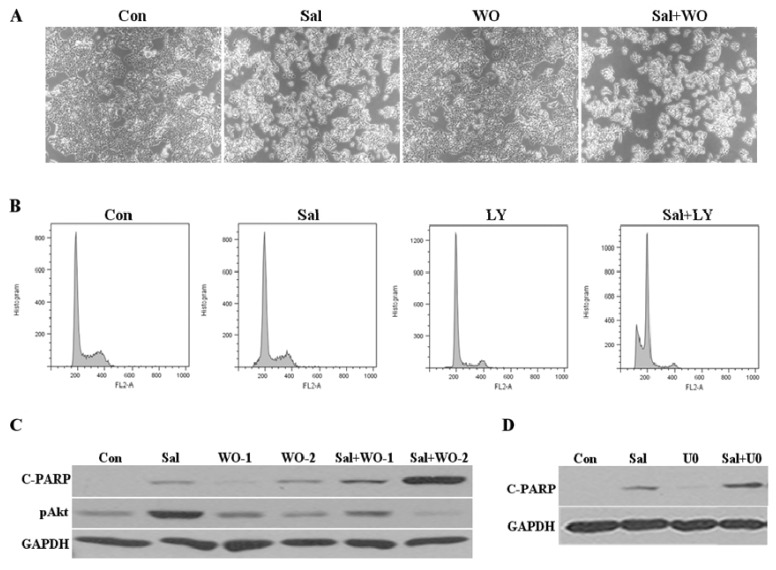
Co-treatment with Akt inhibitor sensitizes Sal-treated cells. (**A**) Hs578T cells were grown on 6-well plates and treated for 48 h with 0.5 μM Sal (Sal), 1 μM wortmannin (WO), 0.5 μM Sal with 1 μM wortmannin (Sal + WO) or DMSO (Con). After 48 h, all cells were observed using an inverted microscope with a 5× objective lens. (**B**) Hs578T cells were grown on 6-well plates and treated with 0.5 μM Sal (Sal), 20 μM LY294002 (LY), 0.5 μM Sal with 20 μM LY294002 (Sal+LY), or DMSO (Con). After 72 h, FACS analysis was performed as described in the Materials and methods. (**C**) Hs578T cells were grown on 6-well plates and treated for 48 h with 0.5 μM Sal (Sal), 1 μM wortmannin (WO-1), 2 μM wortmannin (WO-2), 0.5 μM Sal with 1 μM wortmannin (Sal + WO-1), 0.5 μM Sal with 2 μM wortmannin (Sal + WO-2), or DMSO (Con). After 48 h, the cells were used for western blot analysis using antibodies against C-PARP, pAkt, and GAPDH. (**D**) Hs578T cells were plated on 6-well plates and grown to 30%–40% confluence. The cells were then stimulated for 24 h with 0.5 μM Sal (Sal), 10 μM U0126 (U0), 0.5 μM Sal with 10 ìM U0126 (Sal + U0), or DMSO-treated (Con).

To better understand the roles of Akt activation in cell growth, we investigated the effect of Akt activation in Sal-treated cells in the presence and absence of an Akt inhibitor. After 24 h or 48 h of co-treatment with Sal and the Akt inhibitors, microscopic observation revealed that the co-treatments reduced the cell numbers compared to cells treated with LY294002, wortmannin, or Sal alone (Figure 4A and Figure A2A). Co-treatment with LY294002 also led to apoptosis as shown by the increased C-PARP production and size of the pre-G1 region in the Sal-treated cells (Figure 4B and Figure A2B). We also confirmed that co-treatment with wortmannin increased C-PARP production in Sal-treated cells (Figure A2C). These results indicate that co-treatment of Sal with an Akt inhibitor increases apoptosis compared to cells treated with Akt inhibitors, or Sal alone. Collectively, the results suggest that Akt activation by Sal negatively correlates with Sal cytotoxicity, further suggesting that Akt activation contributes to Sal resistance.

Additionally, we examined whether co-treatment with other kinase inhibitors increases apoptosis in Sal-treated cells. We used the specific well-known kinase inhibitors SP600125 (Jnk), U0126 (Erk), PD98059 (Erk), SB203580 (p38), and AG490 (Jak) [28,30–32]. We performed Western blot analysis to determine the C-PARP production following Sal co-treatment with the inhibitors. When we compared the results with those obtained using LY294002, we found that LY294002 induced the greatest C-PARP production (Figure 4C and Figure A2D), thereby suggesting that Sal co-treatment with an Akt inhibitor has a greater apoptotic effect on cancer cells.

### 2.7. Discussion

Previous reports have shown that Akt activation has a beneficial effect on cancer cells, such as apoptotic resistance by drugs [24]. In the present study, we revealed that Akt activation is significantly increased in cancer cells exposed to Sal. Our data clearly demonstrate that Akt activation by Sal negatively correlates with Sal cytotoxicity, further suggesting that Akt activation contributes to Sal resistance. The results were obtained with low concentrations of Sal because reduced toxicity is important to prevent damage to normal cells when administered to patients. Previously, we have shown that the Sal-sensitization effect can be achieved at low concentrations [26]. The results from this study are important for identifying the signaling pathways and proteins involved in the Sal-sensitization mechanisms at low concentrations.

The finding that co-treatment with PI3K inhibitors inhibited the Sal effect suggests that Sal activates Akt via the PI3K pathway. Although we demonstrated that Sal activates Akt via the PI3K pathway, we did not detect significant changes in the expression levels of the up-stream and down-stream targets and members of the PI3K/Akt/mTOR pathways. We assumed that either very weak activation or very early activation of upstream kinases might increase the Akt activation. Another possibility is that other kinase members or PI3K isoforms upstream of Akt, which were not included in our analysis, might be involved. Therefore, more detailed analysis is required for complete understanding of the Akt activation pathways induced by Sal.

We identified important signal and novel proteins involved in the Sal sensitization. Initially, we determined that Sal significantly reduces p70S6K activation. We conclude that Sal sensitization involves reduced p70S6K activity among the mTOR members and inhibition of cancer cell proliferation; although high levels of Akt activation were observed. The results suggest that Sal sensitization inhibits the mTOR pathway. Previously, we reported that Sal sensitization involves reduced survival rates [16]. Here, we also identified a novel Sal-sensitization mechanism that involves the reduction of the potential transcriptional factor FOXO1 for the survivin expression.

Sal increased the levels of Akt activation in the oral squamous cancer cell line KB and KBV20C cancer cells. In addition, the changes in the signaling proteins in the KB cells had similar patterns to those observed in the Hs578T cells. The results suggest that the Akt pathway is generally conserved in different cancer cell lines. Our results also indicate that Akt activation is independent of MDR phenotype, since both the KB and KBV20C cell lines had similar levels of Akt activation. The activation of Akt was also abolished by the Akt inhibitor, thereby suggesting that the PI3K pathway is also required for Sal-mediated Akt activation both in KB and KBV20C cell lines. Therefore, we demonstrated that Sal treatment increased Akt activation in cancer cell lines originating from a different organ.

Finally, when we analyzed the role of Akt activation, we found that the Akt activation contributes to the reduction of Sal-induced apoptosis. Since co-treatment with Sal and the Akt inhibitor showed a synergistic apoptotic effect. The observations are consistent with the suggestion that cell survival following Sal exposure involves Akt activation-mediated resistance to Sal cytotoxicity. A further suggestion is that Akt activation contributes to Sal resistance. We also showed that co-treatment of an Akt inhibitor with Sal had the greatest apoptotic effects among the tested kinase inhibitors, thereby suggesting that the combination of Sal and an Akt inhibitor would be the best choice to induce increased apoptosis. These results could help to determine the potential clinical use of Sal for cancer patients. For example, the sensitization effects of Sal for clinical applications can be achieved with relatively low concentrations of Sal and Akt inhibition together, thereby avoiding the generation of resistant cancer cells as a result of increased Akt activation. Further studies could examine whether other PI3K inhibitors or Akt inhibitors, which are currently used clinically, can increase sensitization in Sal-treated cancer cells or whether high concentrations of Sal employ a different sensitization mechanism.

## 3. Experimental Section

### 3.1. Reagents

Sal and SP600125 were purchased from Sigma-Aldrich (St. Louis, MO, USA). AG490, LY294002, PD98059, SB203580, and U0126 were supplied by Calbiochem (Bellerica, MA, USA). Wortmannin was supplied by Selleckchem (Houston, TX, USA).

### 3.2. Antibodies

Antibodies against Akt, phosphorylated Akt, phosphorylated IkappaB kinase (IKK)α/β, phosphorylated Jak2, phosphorylated PI3K, PI3K, cleaved poly ADP ribose polymerase (C-PARP), phosphorylated PDK1, phosphorylated GSK3β, phosphorylated p70S6K, PTEN, FOXO1, and phosphorylated Erk1/2 were from Cell Signaling Technology (Danvers, MA, USA). Antibodies against glyceraldehyde 3-phosphate dehydrogenase (GAPDH), phosphorylated p38, p38, Erk1/2, Jak1, Jak2, survivin, and pRb were from Santa Cruz Biotechnology (Santa Cruz, CA, USA). Antibodies against Jnk1, phosphorylated Jnk1, phosphorylated c-Src, and phosphorylated Jak1 were from Biosource (Camarillo, CA, USA). Antibodies against phosphorylated mTOR and phosphorylated PTEN were from Abcam (Cambridge, UK).

### 3.3. Cell Culturing

Hs578T breast cancer cells were obtained from the Korean Cell Line Bank (Seoul, Korea), and were previously used [14,16,17,26]. Human oral squamous carcinoma cell lines, KB and its multidrug-resistant subline, KBV20C, were obtained from Dr. Yong Kee Kim, and they were previously described [29]. All cell lines were cultured in DMEM or RPMI 1640 containing 10% fetal bovine serum, 100 U/mL penicillin, and 100 μg/mL streptomycin (WelGENE, Daegu, Korea).

### 3.4. Western Blot Analysis

All cellular proteins were extracted using a previously described trichloroacetic acid (TCA) method [33,34]. Briefly, proteins were pelleted by centrifugation after addition of 20% TCA and resuspended in 1 M Tris-HCl (pH 8.0). The proteins were subjected to Western blot analysis as described previously [33,34].

### 3.5. Fluorescence-Activated Cell Sorting (FACS) Analysis

FACS analysis was performed as previously described [14,16]. Cells were grown in 60-mm dishes and treated with the indicated drugs for the prescribed times. The cells were then dislodged by trypsin and pelleted by centrifugation. The pelleted cells were washed thoroughly with PBS, suspended in 75% ethanol for at least 1 h at 4 °C, washed again with PBS, and re-suspended in a cold propidium iodide (PI) staining solution (100 μg/mL RNase A and 50 μg/mL PI in PBS) for 40 min at 37 °C. The stained cells were analyzed for relative DNA content using a FACSCalibur flow cytometry system (BD Bioscience, Franklin Lakes, NJ, USA). We performed more than two independent tests.

## 4. Conclusions

The present study enhances our understanding of Sal-sensitization mechanisms. The results were focused on cancer cells in the presence of low concentrations of Sal, and found that Sal-sensitization involves increased levels of Akt and decreased levels of p70S6K activation. Our findings may contribute to the development of Sal-based therapies for patients.
